# Supplementary figures and images for: Distinct pathways for evolution of enhanced receptor binding and cell entry in SARS-like bat coronaviruses
Source: PLoS Pathog. 2024 Nov 15;20(11):e1012704. doi: 10.1371/journal.ppat.1012704 (PMC11602109; doi:10.1371/journal.ppat.1012704)

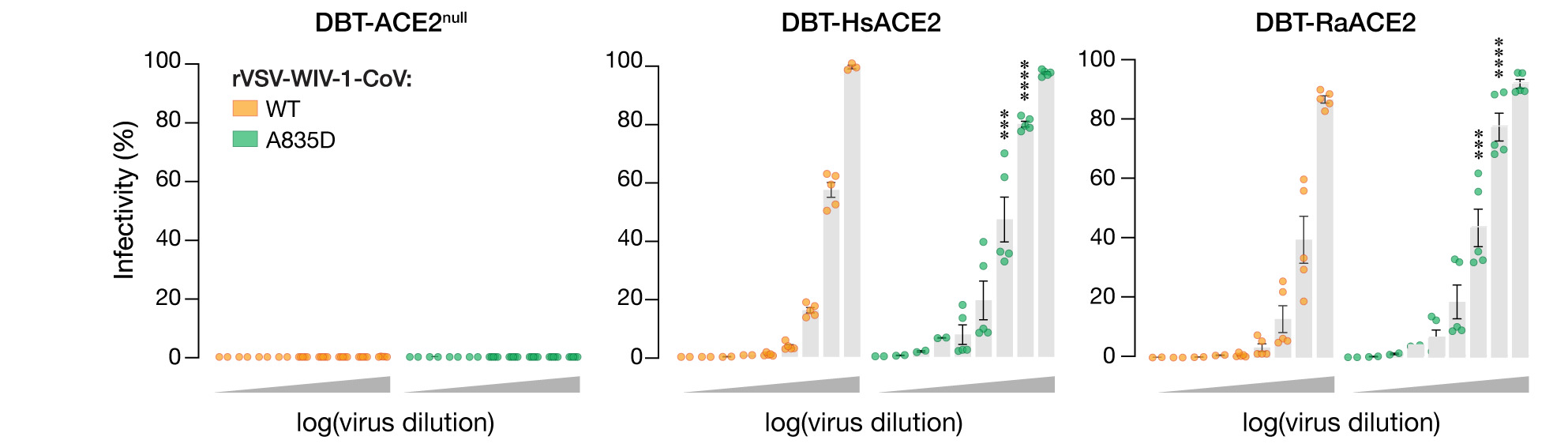

Supplement: S1 Fig — Parental DBT-9 cells or DBT-9 cells overexpressing HsACE2 or RaACE2 were infected with rVSV-WIV-1-CoV WT or A835D particles. Infection was scored by eGFP expression at 12 hours post-infection (average±SD, n = 2–5 from 3 independent experiments). Groups (WT vs. mutant for each cell line) were compared with Welch’s t-test with Holm-Šídák correction for multiple comparisons, ns p>0.05; ** p<0.01; *** p<0.001; **** p<0.0001. Only the statistically significant comparisons are shown. (TIF) [file ppat.1012704.s001.tif]

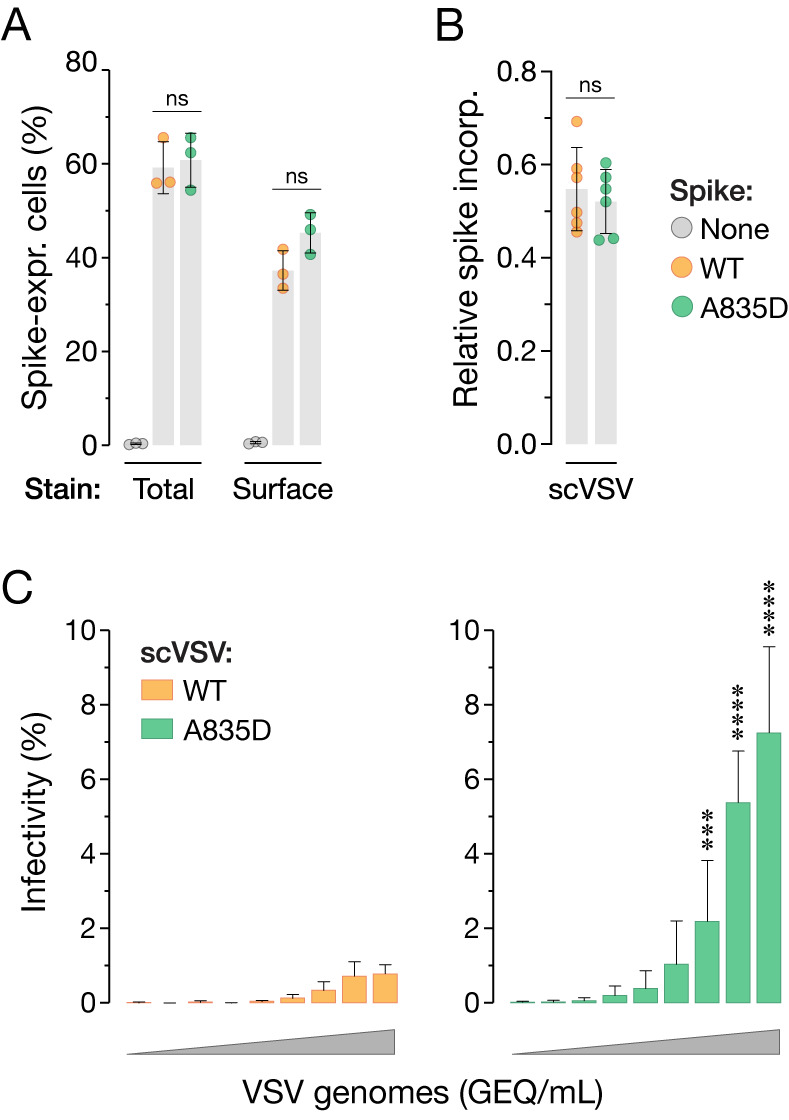

Supplement: S2 Fig — (a) 293FT cells were transfected with plasmids expressing WT or A835D SHC014-CoV spike and immunostained for total protein expression (left) or cell surface expression (right) 24 hours post transfection by a spike-specific antibody and analyzed using flow cytometry (average±SD, n = 3). Groups were compared by one-way ANOVA. (b) scVSV-SHC014-CoV S WT or A835D particles were coated on ELISA plates and probed with a spike-specific mAb or VSV M-specific mAb. The spike-to-M ratio demonstrates VSV particle-specific spike incorporation (average±SD, n = 6 from 3 independent experiments). Groups were compared by one-way ANOVA. (c) Vero cells were infected with pre-titrated amounts of scVSV-SHC014-CoV S WT or A835D. Infection was scored by eGFP expression at 16–18 hours post-infection (average±SD, n = 10–11 from 3 independent experiments). A range of 3.18×101 to 1.24×10^7^ viral GEQ was used. Groups were compared with Welch’s t-test with Holm-Šídák correction for multiple comparisons. ns p>0.05; ** p<0.01; *** p<0.001; **** p<0.0001. In panel c, only the statistically significant comparisons between WT and A835D are shown. (TIF) [file ppat.1012704.s002.tif]

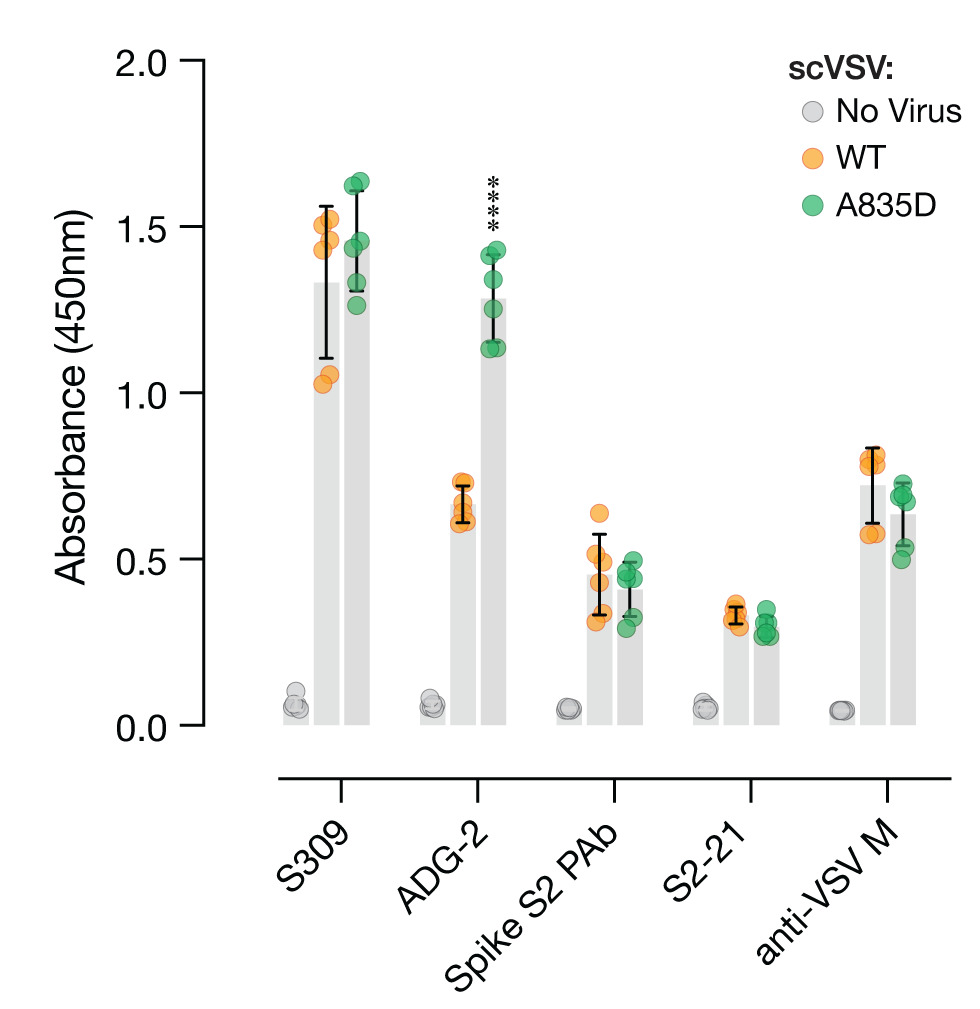

Supplement: S3 Fig — Genome normalized amounts of scVSV-SHC014-CoV S WT and A835D were coated on an ELISA plate and detected with a panel of antibodies, followed by HRP-conjugated secondary antibody. 1.6×10^6^ viral GEQ was used per well. Groups (WT vs. mutant) were compared with two-way ANOVA with Tukey’s correction for multiple comparisons, ns p>0.05; ** p<0.01; *** p<0.001; **** p<0.0001. Only the statistically significant comparisons are shown. (TIF) [file ppat.1012704.s003.tif]

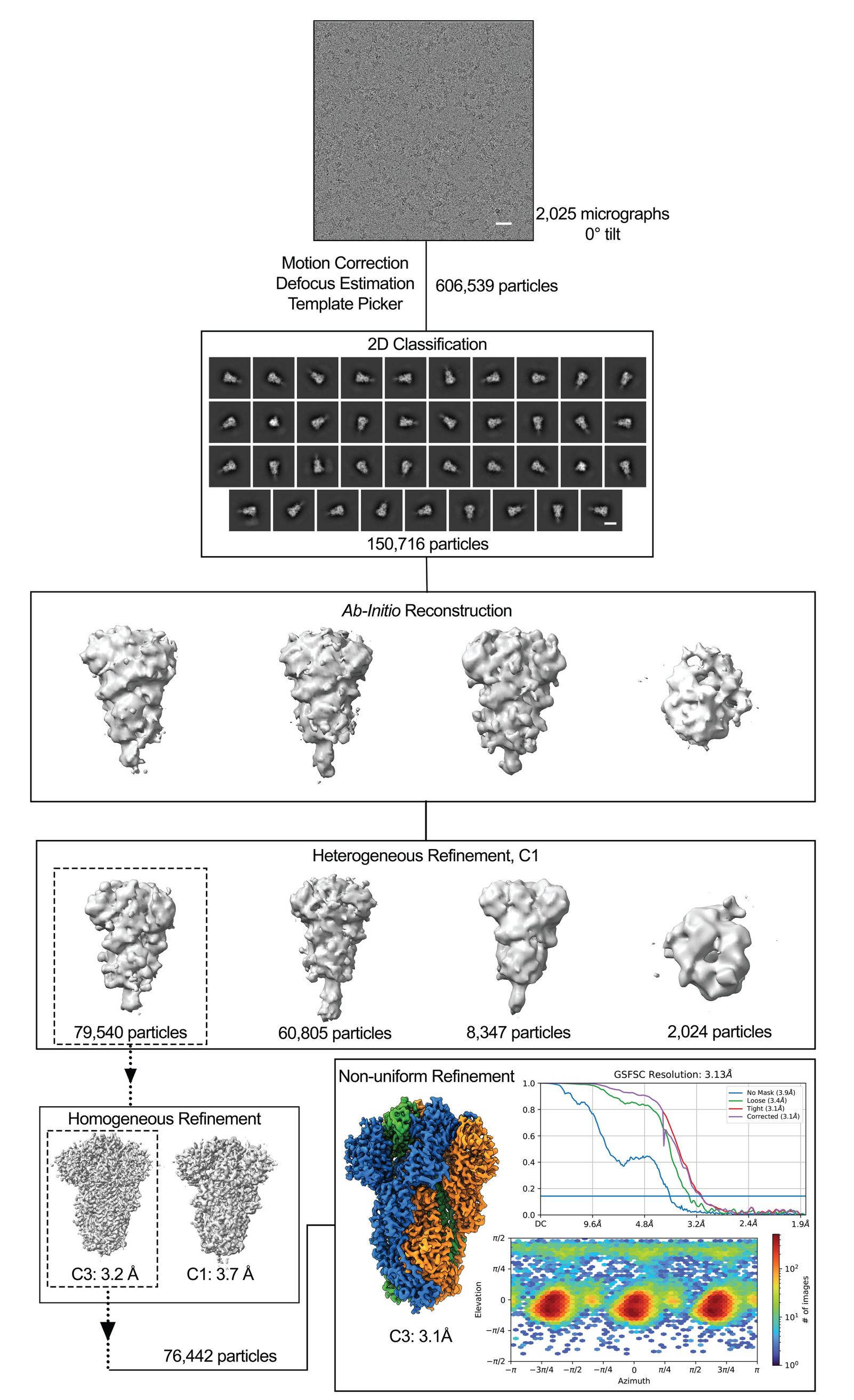

Supplement: S4 Fig — Cryo-EM processing workflow for the WT SHC014-CoV spike. The indicated steps were conducted using cryoSPARC v3: motion correction, contrast transfer function (CTF) estimation, 2D classification, heterogenous refinement, homogenous refinement with C3 symmetry imposed and non-uniform refinement with C3 symmetry imposed. Scale bars in micrograph and 2D classes represent 10 nm. (TIF) [file ppat.1012704.s004.tif]

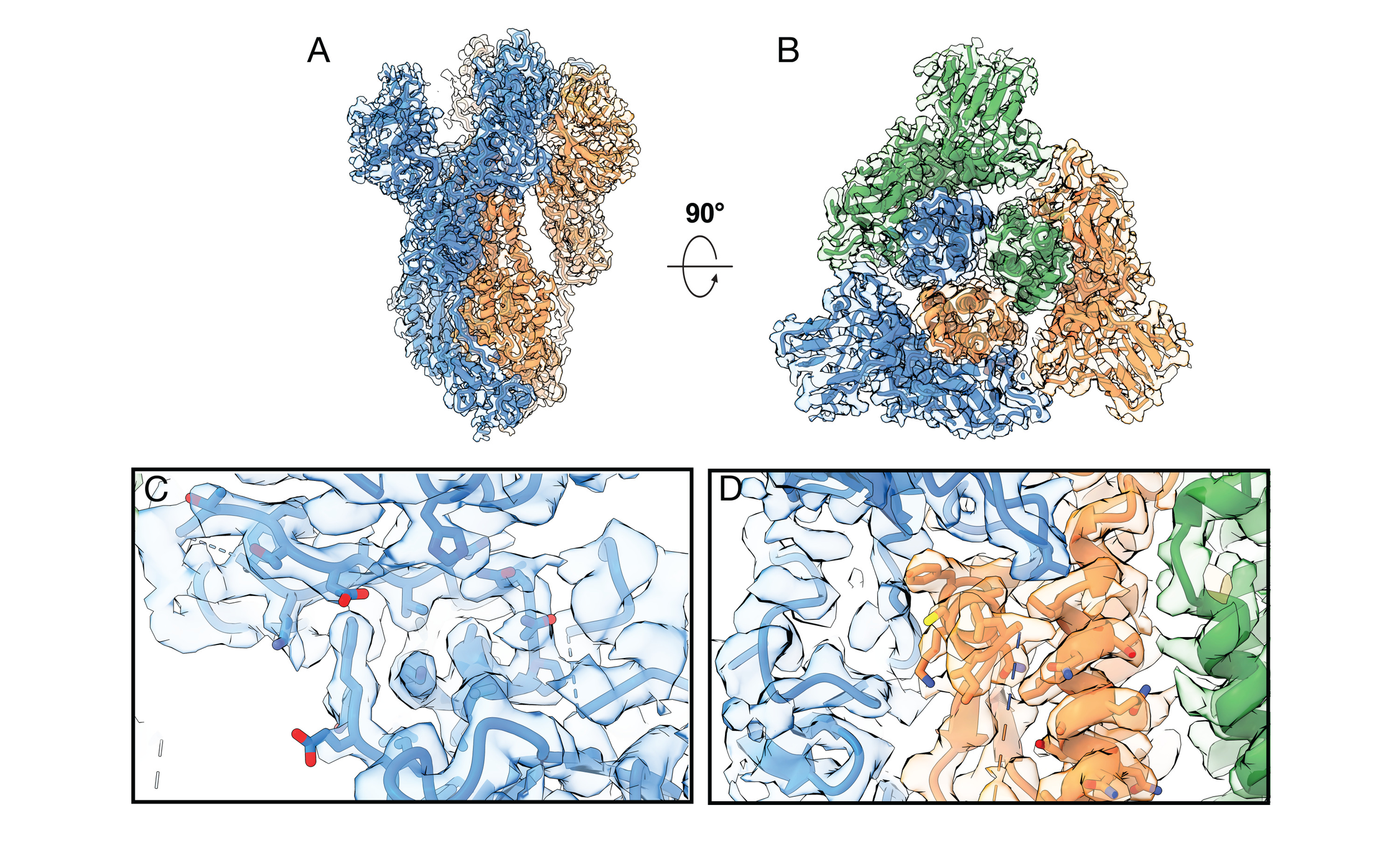

Supplement: S5 Fig — Fit of the SHC014-CoV S model to the refined cryo-EM map. (a) Global map-to-model fit shown with one protomer hidden for clarity. (b) Global map-to-model fit z-slice with all protomers shown. (c) Local fit with side chains shown surrounding Phe294, with Lys266, Lys288, and Phe294 labelled. (d) Local fit with side chains shown surrounding Ala835, with Val556, Ala835, and Val946 labelled. For all panels, a threshold value of 0.19 was used to visualize the cryo-EM map. (TIF) [file ppat.1012704.s005.tif]

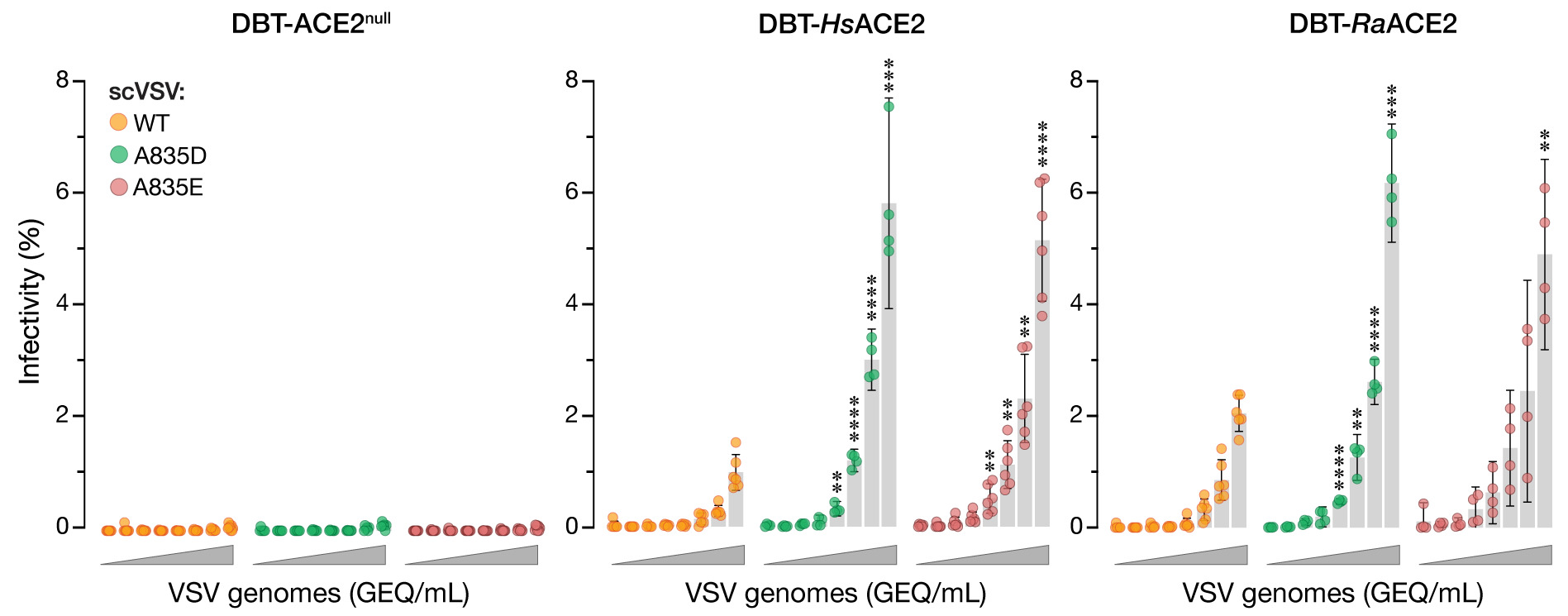

Supplement: S6 Fig — Parental DBT-9 cells or DBT-9 cells overexpressing HsACE2 or RaACE2 were infected with pre-titrated amounts of scVSV-SHC014-CoV particles bearing WT, A835D, or A835E spike. Infection was scored by eGFP expression at 16–18 hours post-infection (average±95%CI, n = 4–8 from 2–3 independent experiments). A range of 4.6×10^2^ to 1.0×10^6^ viral GEQ was used. Groups (WT vs. mutant for each cell line) were compared with Welch’s t-test with Holm-Šídák correction for multiple comparisons. ns p>0.05; ** p<0.01; *** p<0.001; **** p<0.0001. Only the statistically significant comparisons are shown. (TIF) [file ppat.1012704.s006.tif]

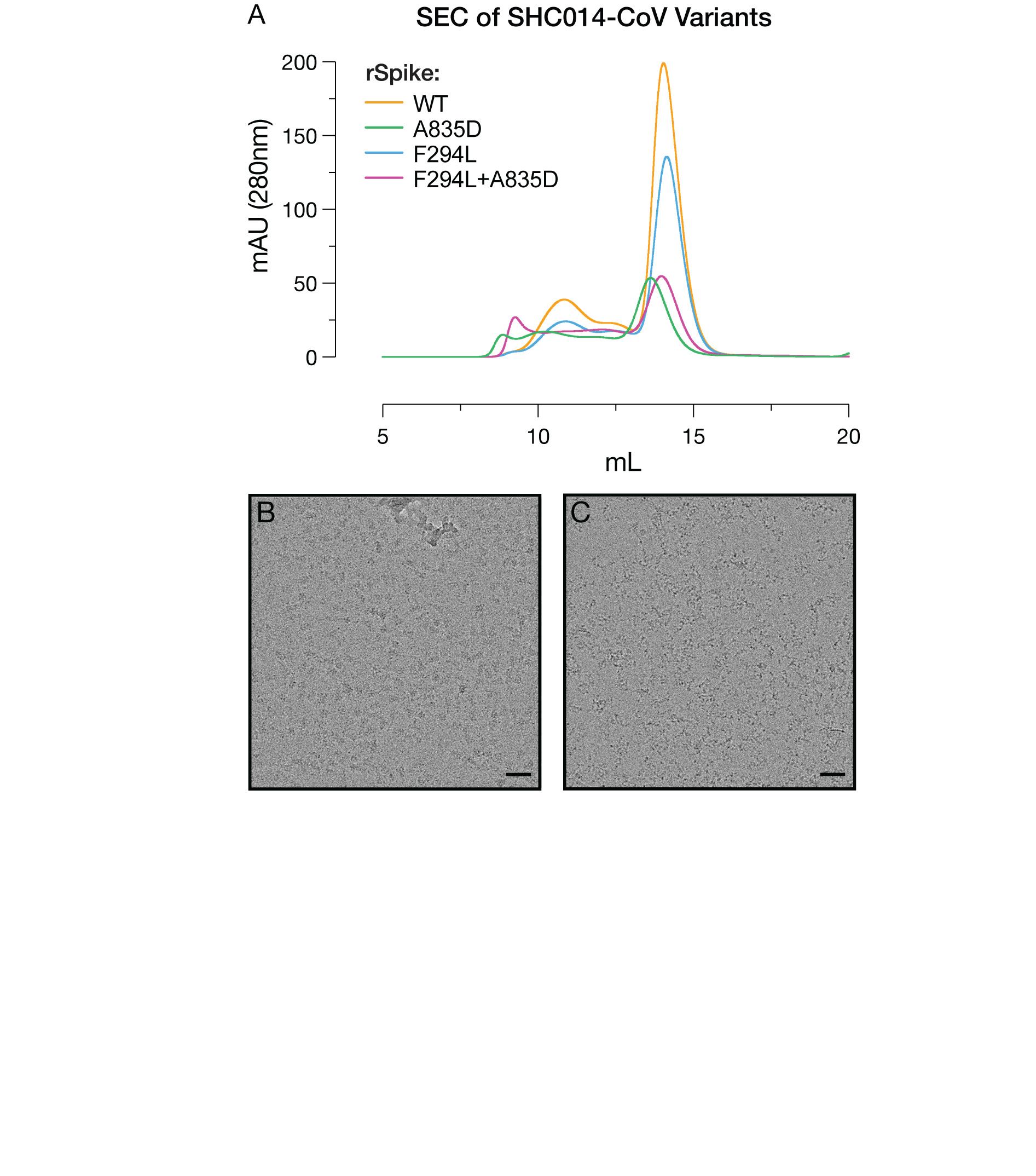

Supplement: S7 Fig — (a) Overlaid size-exclusion chromatography Abs280nm traces show relative protein yield. WT, A835D, F284L, and F294L+A835D SHC014-CoV spikes were expressed and purified in tandem from the same cell line in the same culture volume. Area under the curve represents protein expression. A835D-containing variants exhibit a large decrease in protein expression relative to the WT and F294L SHC014-CoV spikes. (b) WT SHC014-CoV S visualized in a cryo-EM micrograph embedded in the vitreous ice. (c) A835D SHC014-CoV visualized in a cryo-EM micrograph, prepared using the same protocol as in (a). Both spike proteins were expressed and purified at the same time using the same procedures. Scale bars in both represent 10 nm. (TIF) [file ppat.1012704.s007.tif]

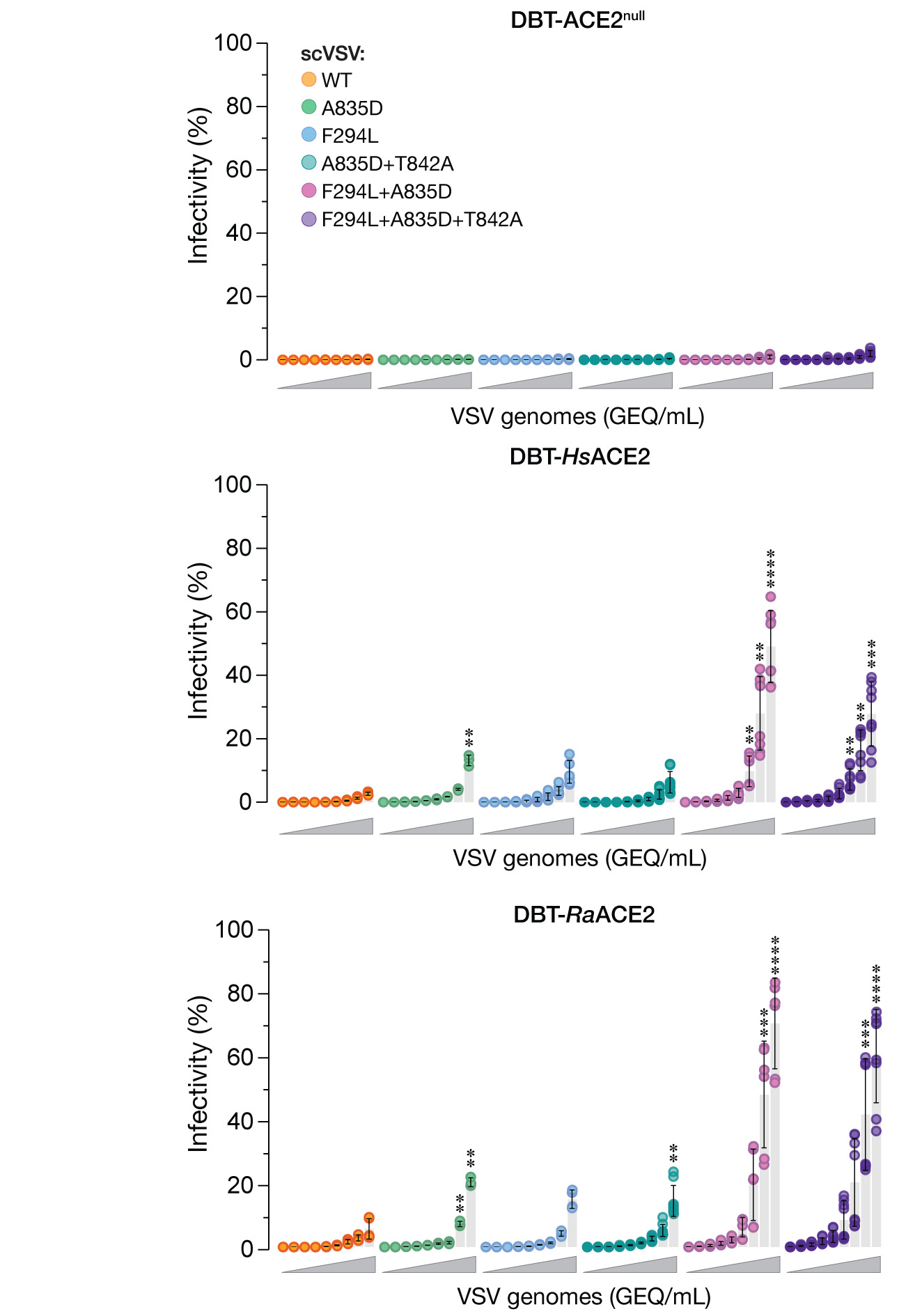

Supplement: S8 Fig — Parental DBT-9 cells or DBT-9 cells overexpressing HsACE2 or RaACE2 were infected with pre-titrated amounts of scVSV-SHC014-CoV particles bearing WT, A835D, F294L, A835D+T842A, A835D+F294L, or F294L+A835D+T842A spike. Infection was scored by eGFP expression at 16–18 hours post-infection (average±95%CI, n = 3–9 from 2–3 independent experiments). A range of 7.15×10^2^ to 4.7×10^6^ viral GEQ was used. Groups (WT vs. mutant for each cell line) were compared with Welch’s t-test with Holm-Šídák correction for multiple comparisons. ns p>0.05; ** p<0.01; *** p<0.001; **** p<0.0001. Only the statistically significant comparisons are shown. (TIF) [file ppat.1012704.s008.tif]

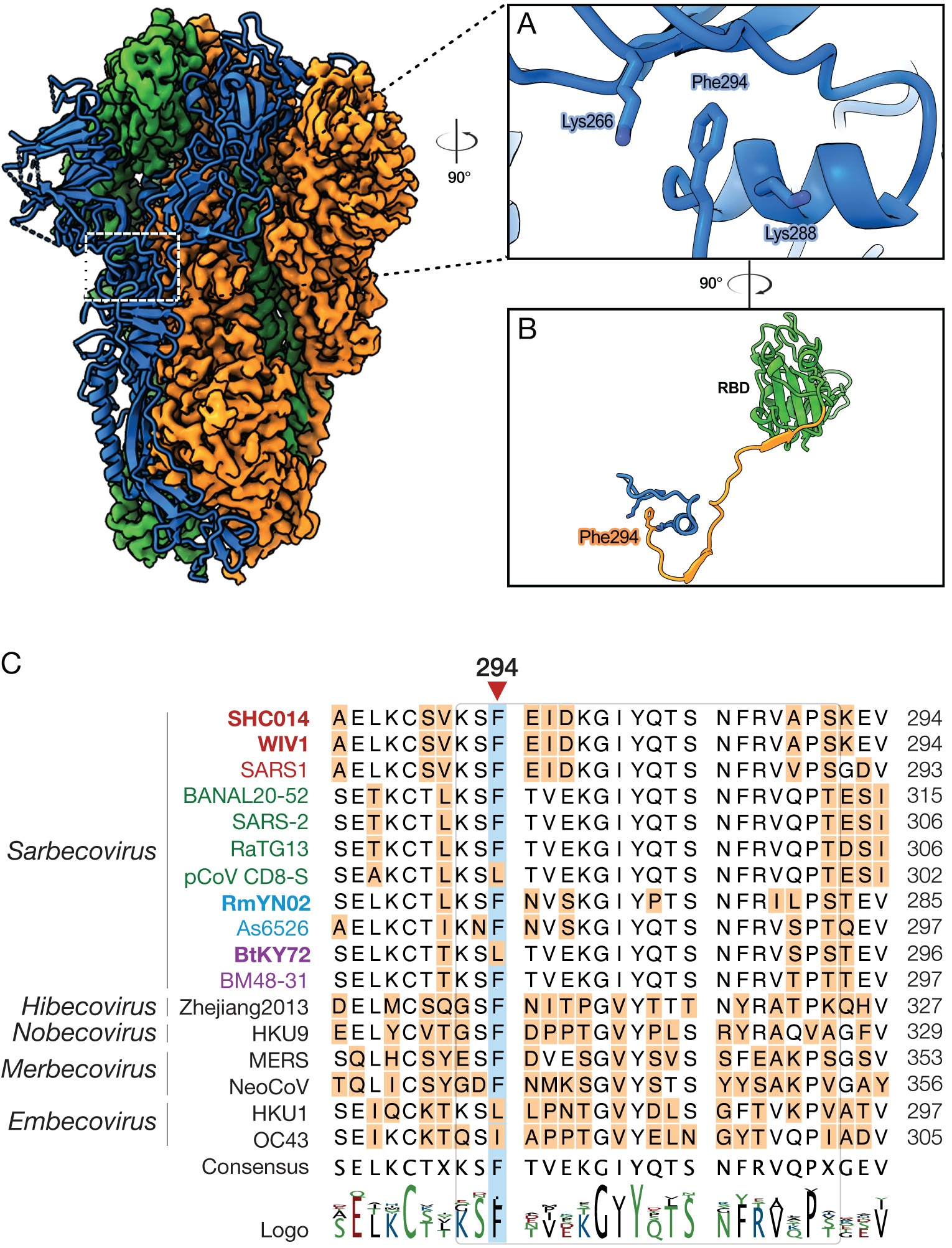

Supplement: S9 Fig — (a) The aromatic ring of Phe294 is positioned between Lys266 and Lys288. (b) The distance between Phe294 and the RBD is depicted, with NTD residues and residues after SD1 hidden for clarity. Labelled residues are modeled as spheres. (c) Alignment of amino acid sequences in the F294 region (rounded rectangle) for selected coronavirus spike proteins. Subgenera are indicated in italics. Sarbecoviruses are color-coded by clade (1a: SARS-CoV–like, red; 1b: SARS-CoV-2–like, green; 2: Southeast Asian bat-origin CoV, blue; 3: non-Asian bat-origin CoV, purple). Spikes investigated in the current study are in bold. (TIF) [file ppat.1012704.s009.tif]
