# Supplementary material for: Distinct pathways for evolution of enhanced receptor binding and cell entry in SARS-like bat coronaviruses
Source: PLoS Pathog. 2024 Nov 15;20(11):e1012704. doi: 10.1371/journal.ppat.1012704 (PMC11602109; doi:10.1371/journal.ppat.1012704)
Supplement: S1 Table — (DOCX) [file ppat.1012704.s010.docx]

| **EM data collection** |  |
| --- | --- |
| Sample | Bat SARS-like CoV RsSHC014-CoV S |
| Voltage (kV) | 200 |
| Detector | Falcon 4 |
| Magnification (nominal) | 105,000 |
| Pixel size (Å/pix) | 0.94 |
| Exposure rate (e^-^/pix/sec) | 3.40 |
| Frames per exposure | 50 |
| Exposure (e^-^/Å^2^) | 50 |
| Defocus range (µm) | 1.0-2.0 |
| Tilt angle ( ̊ ) | 0 |
| Micrographs collected | 2,025 |
| Micrographs used | 1,097 |
| Particles extracted (total) | 419,182 |
| Automation software | SerialEM |
| **3D reconstruction statistics** |  |
| Particles | 76,442 |
| Symmetry | C3 |
| Map sharpening B-factor | -82.8 |
| Unmasked resolution at 0.5 FSC (Å) | 7.5 |
| Masked resolution at 0.5 FSC (Å) | 3.7 |
| Unmasked resolution at 0.143 FSC (Å) | 3.9 |
| Masked resolution at 0.143 FSC (Å) | 3.1 |
| **Model refinement and validation statistics** |  |
| Refinement package | Phenix |
| Composition |  |
| Amino acids | 2,925 |
| RMSD bonds (Å) | 0.005 |
| RMSD angles (º) | 0.98 |
| Average B-factors |  |
| Amino acids | 71.2 |
| Ramachandran |  |
| Favored (%) | 96.0 |
| Allowed (%) | 4.0 |
| Outliers (%) | 0.0 |
| Rotamer outliers (%) | 0.47 |
| Clash score | 5.84 |
| C-beta outliers (%) | 0 |
| CaBLAM outliers (%) | 3.12 |
| CC (mask) | 0.78 |
| MolProbity score | 1.59 |
