# Supplementary material for: Distinct pathways for evolution of enhanced receptor binding and cell entry in SARS-like bat coronaviruses
Source: PLoS Pathog. 2024 Nov 15;20(11):e1012704. doi: 10.1371/journal.ppat.1012704 (PMC11602109; doi:10.1371/journal.ppat.1012704)
Supplement: Attachment — Submitted filename: Tse_Response to Reviewers.docx [file ppat.1012704.s014.docx]

We thank the editor and reviewers for their clear guidance and valuable feedback, which we have incorporated into the revised manuscript.

**Editor’s Comments**

I also suggest (as also alluded by Reviewer 2) that you add a bit more discussion of studies of similar phenomenon in SARS-CoV-2 evolution, and how it relates to what you observe. In particular, there is clear experimental evidence that SARS-CoV-2 spike has fixed mutations that have modulate RBD conformation during its evolution in humans, and also that mutations that alter RBD conformation affect serum antibody neutralization. See for instance Zhang et al, Molecular Cell, 84:2747 and Dadonaite et al, Nature, 631:617.

We have expanded the discussion on SARS-CoV-2 evolution as suggested.

Please introduce subgenus and genus taxa, according to virus taxonomy rules, e.g. replace "...isolated seven distinct full-length CoV sequences belonging to the subgenus of SARS-like betacoronaviruses (sarbecoviruses), including two from novel agents— Rs3367 and RsSHC014—and determined their full-length genome sequences" with "...determined full-length genomic sequences of seven CoVs belonging to the subgenus *Sarbecovirus*, genus *Betacoronavirus*, including two from novel agents — Rs3367 and RsSHC014."

Please introduce phylogenetic clades used in Fig. 1 and elsewhere; note, they may be prone to intra-species homologous recombination.

We have modified the text and Fig 1c legend to introduce the recommended changes.

**Reviewer 1:**

Major Issues

1. Throughout the manuscript, the authors discuss epistasis and/or synergy between two mutations that enhance RBD opening (A835D and F294L). They correctly define positive epistasis as “a phenomenon in which the combined effect of two or more mutations in a protein is greater than (and/or distinct from) the sum of their individual effects”, but I’m not sure they apply this proper definition to the interpretation of their data. The current metrics for evaluating epistasis are a bit more qualitative than quantitative (i.e. visual comparison of plots in Figure 6) which complicates the identification of epistasis as a quantitative deviation from additive expectation, but for example in Figure. 6b, I do not see clear evidence of epistasis: F294L enhances viral infectivity, A835D enhances viral infectivity, and the double mutant has even further enhanced infectivity – as would be expected if the two mutations were additive. To truly prove synergistic epistasis, the authors would need (1) to define a quantitative metric to summarize each genotype’s phenotype; (2) prove the background scale on which mutations are expected to combine additively (e.g. addition of a mutation with effect +1 and another with +2 yields expected double mutant phenotype of +3 on a linearly additive scale (so epistasis is present if phenotype != +3), but some phenotypes are multiplicative e.g. a mutation with a 1.1-fold effect and another with a 1.2-fold effect would be expected to have a 1.32-fold effect when combined; and yet other phenotypes are simply nonlinear even in the absence of epistasis, e.g. diminishing returns epistasis), and (3) show that the double mutant deviates from the non-epistatic expectation. Synergy means that there is more increase in infectivity than expected from single mutant measurements, not simply that the double mutant has stronger infectivity than either single mutant alone. In fact, the authors own language points to the non-epistatic relationship between these two mutations’ effects, p. 17: “F294L+A835D conferred a striking **additive** enhancement in infectivity relative to the single mutants”.

We are grateful to the reviewer for pointing out the lack of quantitative rigor in our initial description of an epistasis between F294L and A835D. We have addressed this through the use of a published linear model-based approach to analyze our infectivity and ACE2 binding datasets. This analysis, now included in S2 Table, indicates that the non-additive effect of combining the two mutations approaches, but does not reach, statistical significance. We believe that the possibility of epistasis still exists but will require a more comprehensive mutagenesis approach to uncover. We have modified the manuscript throughout to address and discuss these new findings.

Minor Issues

1. The authors do discuss the T372A mutation underlying SARS-CoV-2 emergence in the discussion, but I found its absence notable in the introduction. This mutation has been shown to enhance replication and suggested to underlie SARS-CoV-2’s propensity for RBD opening relative to bat sarbecoviruses like RsSHC014 that pack their RBDs down more frequently. T372A probably has an incredibly important role in SARS-CoV-2’s transmissibility and therefore pandemic spread, and given it’s likely a related mechanism to what they authors discover and characterize in more detail with their A835D mutation in RsSHC014, the relevance of this mechanism could be emphasized more in relation to pandemic virus emergence.

The revised manuscript now discusses T372A in the Introduction and elaborates on its role in SARS-CoV-2 adaptation in the Discussion.

**Reviewer 2:**

Minor Issues

1. While the use of the ADG2 antibody sufficiently supports the conclusions drawn in the paper regarding epitope accessibility, binding studies performed with a panel of antibodies targeting different regions of the spike protein would be a valuable addition as it would reveal any allosteric effects of the FPPR mutation on other regions of the spike. How the FPPR mutation impacts S2 directed epitopes would be of interest, for example.

We appreciate the reviewer’s suggestion. We have conducted additional binding studies using S1 and S2-directed antibodies against scVSV-SHC014-CoV WT and A835D particles to investigate any allosteric effects of the FPPR mutation and have included these data in a new supplemental figure, S3 Fig.

2. This sentence in the discussion “Recent work suggests that, in contrast to SARS-CoV-2, multiple pre-fusion spikes from CoVs of rhinolophid bat and pangolin origin (e.g., BANAL-20-52, BANAL-20-236, RaTG13, Pangolin-CoV), have evolved to favor a more compact ‘closed’ state,” should be modulated/qualified to recognize that the SARS-CoV-2 Omicron variant has been shown to increasingly favor “closed” states.

We have modified the text to include this qualifier.

3. While the structural conclusions are well supported by the experimental data, it may be worth adding a note that a larger dataset and particle number may have enabled more extensive classification to reveal any small population of up states that may be present. While this would not change any of the conclusions drawn in the paper, to a non-structural, general reader, it would provide an understanding of the methodological caveats in a cryo-EM experiments.

We have added language to the text to address this point.

4. In this sentence “Disruption of RBD-FPPR interaction in the A835D mutant is predicted to free the linker to contribute to the RBD movements associated with spike opening.” The phrase “Disruption of RBD-FPPR interaction” may be confusion as there is no direct interaction between RBD and FPPR. Consider rephrasing. Perhaps “allostery” may be a more suitable word.

Given the lack of strong statistical evidence for non-additive effects of the F294L and A835D substitutions (see response to Reviewer 1 point 1), we believe this sentence, which implies a mechanistic synergy between the substitutions, to no longer be appropriate and have removed it from the revised manuscript.

5. In the section titled “A835D does not alter spike expression or VSV incorporation.”, the references to the Supplemental Figures need to be corrected. For example, Figure S2c does not show ELISA data as referred to in the text.

The references to Supplemental Figure 2 b and c in this section have been corrected.

6. For the Figures reporting infectivity assays, for example Figure 2, the legend should state what reference was used to define 100% infectivity.

In the figures reporting Infectivity (%), including Figure 2, infectivity values were calculated as the ratio of the number of infected cells over total number of cells in each well. We have updated the figure legends to clarify this. Further, for any figures in which values were normalized to a reference value, we have added this information to the figure legends.

**Reviewer 3:**

Major Issues

1. The bat SARSr-CoV SHC014 was initially identified from Rhinolophus sinicus. Why the authors didn’t use Rhinolophus affinis ACE2 in their study? The authors showed that the A835D mutation occurred during the rescue in Vero cells, but subsequent receptor usage assessments and measurements were conducted in DBT-human ACE2 and DBT-Rhinolophus affinis ACE2 cell lines, or with human and Rhinolophus affinis ACE2 proteins. The authors should also test the natural host ACE2 to determine if the mutations are compatible with its host receptor, including binding and infection assays, to assess whether the mutations enhance or diminish its own receptor interaction.

Previous studies have shown that the isolated SHC014-CoV RBD binds with nM affinity to diverse ACE2 orthologs, including from human, civet, pangolin, mouse, intermediate horseshoe bat (*R. affinis*), and Chinese rufous horseshoe bat (*R. sinicus*) (Starr et al., 2022). Indeed, SHC014-CoV RBD binds with higher affinity to both human and *R. affinis* ACE2 compared to ACE2 from *R. sinicus*. These and other data point to the broad ACE2 receptor use by sarbecoviruses (Lee et al., 2023). Most importantly, however, our study is focused on RBD availability/deployment and not RBD-ACE2 affinity. For these reasons, studies with *R. sinicus* ACE2 are unlikely to substantively alter our conclusions.

2. The authors demonstrate “we could recover rVSV-RmYN02-CoV S(A806D) and rVSV-BtKY72-CoV S(A837D)”, but no data is presented. Additionally, since clade 2 SARSr-CoV requires trypsin for recovery, what conditions were used for rVSV-RmYN02-CoV S(A806D) and rVSV-BtKY72-CoV S recovery? Was trypsin added? The authors should clarify these details so that readers can understand whether the mutation is the critical factor.

We have added additional details to Methods describing the recovery of rVSV-RmYN02-CoV S(A806D) and rVSV-BtKY72-CoV S(A837D). Specifically, no trypsin was added to these cultures during rVSV recovery and successful rescues were conducted at 32°C.

3. The authors showed that mutations in the SHC014 spike could shift its RBD from a "down" to an "up" state. Have they tested whether the same mutations in RmYN02-CoV S and BtKY72-CoV S would result in similar structural changes? If structural analysis is challenging, the authors could instead assess their neutralization abilities using different antibodies that recognize different epitopes, as they did with the SHC014 spike. Additionally, if these mutations in RmYN02-CoV S and BtKY72-CoV S also expose their RBD to an "up" state, would they be able to bind human ACE2? This is particularly relevant for BtKY72-CoV S, which naturally binds to its host's ACE2. With these mutations, would their binding affinity to their natural host's ACE2 and human ACE2 improve or decline?

Unfortunately, we were unable to conduct any infectivity or binding assays with scVSV RmYN02-CoV or BtKY72-CoV. Despite being able to rescue these rVSVs with the cognate A835D substitution, the resulting viruses did not grow well, suggesting that one or more additional factor(s) are necessary for sufficient growth to conduct any additional infectivity assays in cell culture. In our scVSV preps, both viruses had poor infectivity levels, also suggesting that there are other barriers to entry for these viruses that have yet to be elucidated. For binding studies, RmYN02-CoV is not predicted to use ACE2 based on previous studies showing lack of binding between soluble RmYN02-CoV RBD and ACE2 proteins (Starr et al., 2022). Additionally, these same studies showed that although predicted to use ACE2, BtKY72-CoV RBD is unable to bind to soluble human ACE2, making binding studies challenging.

4. Why did the authors use the SARS-CoV-2 backbone instead of the SHC014 backbone for the replicon-based system? Would the SHC014 backbone result in better or worse growth with its own spike? The authors should explore this further.

We appreciate the reviewer’s suggestion regarding using a SHC014 backbone for a replicon-based system. However, building such a system is a significant undertaking and beyond the scope of the current manuscript. We do hope to generate such a system for future work.

5. The authors used DBT-RaACE2 cells for the spike thermostability and temperature dependence experiments, while human ACE2 was used for binding experiments. Did the authors also use human ACE2 cells and RaACE2 protein for these tests? If so, are the results consistent? The authors should include these results in the manuscript.

We did not use human ACE2 cells and *Ra*ACE2 protein for thermostability experiments. As discussed in point 3. above, SHC014-CoV RBD binds both *Hs*ACE2 and *Ra*ACE2 with high affinity. Given that the focus of our study is RBD availability, not affinity, we believe that the experiments with *Hs*ACE2 cells and *Ra*ACE2 protein would elicit conclusions substantively similar to those presented.

6. The F294L mutation was observed in the SHC014 spike along with the A835D and T842A double mutations. Why did the authors test F294L only with A835D or alone, but not with both A835D and T842A mutations together?

The revised manuscript now includes a new supplementary figure (S7 Fig) that dissects the role of T842A and demonstrates that it does not contribute to the phenotypes of A835D and F294L. These findings rationalize our omission of T842A in the subsequent rVSV and scVSV studies.

7. In Fig. 6b, the F294L+A835D double mutations infect DBT-ACE2 null cells at higher virus concentration, how to interpret this result? Does it indicate that the SHC014 spike can enter cells independently of ACE2 with these two mutations?

Although there is a statistically significant increase in the infection of DBT-ACE2–null cells at higher virus concentrations, the effect size (i.e., level of infectivity over background) is extremely small and unlikely to represent a biologically meaningful result.

8. The authors tested the antibody sensitivity of the SHC014 spike with mutations using only one antibody that binds to the RBD 'Up' state. They should also test antibodies that specifically recognize the RBD 'Down' state to determine if the mutated spike is less sensitive compared to the WT with these antibodies.

Although this would be an interesting experiment, Unfortunately, we are not aware of any RBD ‘Down’ state-specific antibodies that bind and/or neutralize SHC014-CoV. However, we have now tested a panel of antibodies for binding against scVSV-SHC014-CoV S WT and A835D and included this in a new supplemental figure (S3 Fig). This panel includes S309, an RBD-directed monoclonal antibody that is not dependent on RBD ‘Up’ or ‘Down’ positioning and binds equally well to the WT and A835D spikes.

9. The authors demonstrated that the A835D, F294L, or double mutations in the SHC014 spike enhance human ACE2 binding and increase infectivity in DBT-human ACE2 and Ra ACE2 cell lines. Since infection also involves membrane fusion and A835D is located in the S2 subunit, the authors should investigate whether this mutation affects membrane fusion efficiency, such as changes in protease dependence or fusion ability.

We agree with the reviewer that these are interesting and important experiments. However, given the extensive mechanistic workup already presented, including new experiments performed for the revised manuscript, we feel that these additional studies are beyond the scope of the manuscript. We are currently designing follow-up experiments to investigate these questions.

10. In Fig. S2, did the authors use a specific SARS-CoV-2 spike antibody to measure scVSV-SHC014-CoV S?

Yes, the monoclonal antibody S309 was used for S2 Fig. This information has been added to Methods.

Minor Issues

1. Please uniform the writing of “SARS-like-CoV” and “SARS-related-CoV”; “RsSHC014” “SHC014”, “SHC014 CoV” and “SHC014-CoV”; “SARS-CoV-1” and “SARS-CoV” and so on, throughout the whole manuscript.

We have standardized our nomenclature for these viruses throughout the manuscript.

2. Most figures lack clear labels, making data interpretation difficult without figure legends or the main text. The authors should revise the figures for clarity.

We have added labels to figures to enhance clarity wherever possible.

3. There are no methods described for assessing spike incorporation into viral particles using ELISA

We thank the reviewer for noting this omission. It has now been corrected.

4. In the text “Despite this, scVSVs bearing the A835D spike were much more infectious than their WT counterparts in Vero cells, recapitulating the phenotypes observed in the rVSV and CoV RDP systems (Fig. S2b). ”，Fig.S2b should be Fig.2c.

We thank the reviewer for catching this error and have corrected it in the revised manuscript.

5. In the text “In all, we were able to build residues 33-1124, excluding 45–4, 71–78, 94–97, 121–123, 134–155, 167–182, 205–212, 237251, 268–270, 617–621, 665–671, and 812-831.”, please check “45-4”.

The text has been corrected to read “45-46.”

6. In the methods “Temperature-Dependent Infection Assays” authors described they used RsACE2, please check.

We have corrected the text to correctly describe the use of *Ra*ACE2.

7. The method “ADG-2 Neutralization Assay”, authors used DBT-RaACE2 cells, while in the figure 7e legend, Vero cells were used. Please check.

Thank you for noting this error, DBT*-Ra*ACE2 cells were used for this assay and the figure legend has been corrected.
